# Unhappy 20/20: A New Challenge for Cataract Surgery

**DOI:** 10.3390/jcm14051408

**Published:** 2025-02-20

**Authors:** Chiara De Gregorio, Sebastiano Nunziata, Sara Spelta, Paolo Lauretti, Vincenzo Barone, Pier Luigi Surico, Tommaso Mori, Marco Coassin, Antonio Di Zazzo

**Affiliations:** 1 Ophthalmology Complex Operative Unit, Ophthalmology Campus Bio-Medico University, 00128 Rome, Italy; 2 Ophthalmology Operative Complex Unit, Campus Bio-Medico University Hospital Foundation, 00128 Rome, Italy; 3 Casa di Cura San Marco, 04100 Latina, Italy; 4 Cornea Service, Campus Bio-Medico University Hospital Foundation, 00128 Rome, Italy

**Keywords:** cataract, cataract surgery, dry eye, unhappy 20/20

## Abstract

**Background/Objectives:** Cataract surgery has evolved into a refractive procedure aimed at optimizing both vision quality and quantity. Modern patients, particularly “baby boomers”, expect superior outcomes, increasing demand for premium intraocular lenses (IOLs). However, ocular surface dysfunction (OSD), especially dry eye disease (DED), compromises postoperative satisfaction, with up to 35% of patients dissatisfied despite achieving 20/20 visual acuity. This study aimed to characterize postsurgical ocular surface system failure (OSSF) and explore strategies to improve perioperative management. **Methods:** An open observational study was conducted at the Ophthalmology Complex Operative Unit, University Campus Bio-Medico, Rome, Italy, enrolling 20 patients with stage N2–3 and C1–2 lens opacities. Patients with diabetes, prior surgeries, or ocular inflammatory diseases were excluded. Preoperative and postoperative assessments included OSDI, SANDE scores, Schirmer test, TBUT, and fluorescein staining. Follow-ups occurred at 1 week, 1 month, and 3 months postoperatively. Statistical analysis used two-way ANOVA (*p* < 0.05). **Results:** Despite achieving a BCVA of 20/20, 44% of patients reported OSSF symptoms. Postoperative evaluations revealed significant worsening in OSDI and SANDE scores (*p* < 0.001), Schirmer test (preoperative mean 19.92 ± 10.06; *p* < 0.001), and TBUT (preoperative mean 5.88 ± 2.64 s; *p* < 0.001). Meibomian gland dysfunction and conjunctival hyperemia also worsened. **Conclusions:** Postsurgical OSSF results from neurogenic inflammation, tear film instability, and meibomian gland dysfunction, exacerbated by surgical trauma. Preoperative and postoperative management, including artificial tears, lid hygiene, and preservative-free regimens, are essential to improve outcomes and patient satisfaction. Comprehensive strategies can mitigate symptoms and enhance the benefits of cataract surgery.

## 1. Background

In recent years, cataract surgery has evolved from a functional procedure, primarily aimed at removing the pathological cloudy lens, to a sophisticated refractive intervention. Modern patients undergoing cataract surgery often seek not only the restoration of vision but also enhancements in visual quality to support their active lifestyles. This shift is driven by younger, healthier individuals who are increasingly aware of the initial visual disturbances caused by mild lens opacities and are well informed—though sometimes misinformed—about surgical possibilities [1]. Many of these patients belong to the “baby boomer” generation, born between 1946 and 1967, who often maintain an active lifestyle and delay retirement. Their exposure to online information, which can be both empowering and misleading, adds complexity to preoperative consultations and intraocular lens (IOL) selection processes. For these patients, the success of cataract surgery is measured not only by the restoration of visual acuity but also by subjective improvements in vision quality and overall satisfaction. This shift in patient expectations presents ophthalmologists with the challenge of addressing a broader spectrum of needs, including factors such as contrast sensitivity, glare, and optical aberrations. Achieving optimal outcomes relies on precise IOL power calculations and alignment, as well as the comprehensive management of ocular surface health, which is increasingly recognized as a key determinant of postoperative success.

Ocular surface disease (OSD), particularly dry eye disease (DED), plays a pivotal role in perioperative outcomes. Research indicates that over 80% of patients experience some degree of DED after cataract surgery, 20% have preexisting DED, and 60% remain undiagnosed prior to the procedure. Despite achieving an excellent uncorrected or best-corrected visual acuity (UCVA or BCVA) of 20/20, up to 35% of patients report dissatisfaction due to ocular surface system failure (OSSF) triggered by surgical trauma [2]. This dissatisfaction highlights the multifactorial nature of visual quality, which depends on the interplay between optical, neurological, and ocular surface systems.

The timing, features, and underlying mechanisms of postsurgical OSSF remain poorly understood. Numerous studies have shown that the type of surgery and the surgical techniques used can significantly impact postoperative ocular surface health. For instance, techniques such as phacoemulsification with small-incision surgery have been shown to reduce surgical trauma compared to more invasive techniques like extracapsular cataract extraction (ECCE). Additionally, the adoption of advanced technologies, such as femtosecond laser, offers precision in surgical outcome but added new variables in the determination of postoperative dry eye. Recent studies have also highlighted the crucial role of proper preoperative management, emphasizing that early identification and treatment of preexisting conditions, such as meibomian gland dysfunction or dry eye disease, can improve postoperative outcomes and reduce the risk of ocular surface complications [3,4].

Addressing these gaps is critical, particularly as the prevalence of refractive cataract surgery continues to rise, emphasizing the need for patient-centered outcomes. The aim of our study is to describe the characteristics of ocular surface dysfunction in “unhappy 20/20” patients following cataract surgery, providing insights into its etiology and potential strategies for prevention and management. “Unhappy 20/20” refers to patients who have achieved excellent best-corrected visual acuity (BCVA), typically measured as 0.00 logMAR, following cataract surgery but report dissatisfaction due to poor visual quality. Despite having optimal visual acuity, these patients experience symptoms like glare, halos, contrast sensitivity issues, or ocular discomfort, which significantly impact their overall visual satisfaction and quality of life, like a failure of the ocular surface system, but does not meet the diagnostic criteria for dry eye.

## 2. Methods

Patients were enrolled in an open perspective observational study at the Ophthalmology Complex Operative Unit, University Campus Bio-Medico (Rome, Italy). The study adhered to the ARVO guidelines and the Declaration of Helsinki concerning research involving human subjects. Written informed consent was obtained from all participants before any clinical analysis. Patients included in the study were required to meet the following criteria: age > 18 years, patients scheduled for cataract surgery with lens opacities classified as stage N2–3 and C1–2 according to the Lens Opacities Classification System (LOCS), willingness to provide informed consent, and availability for full participation in all study procedures and follow-ups. Exclusion criteria included the presence of diabetes, use of topical or systemic anti-inflammatory medications, a concomitant diagnosis of glaucoma or antiglaucoma therapy, systemic or local therapy with medications affecting tear film secretion (e.g., beta-blockers or antidepressants), or a history of allergic, congenital, or systemic autoimmune diseases. Patients were also excluded if they had a medical history of ocular or systemic inflammatory, autoimmune, or autoinflammatory diseases, prior ocular surgery, or other concurrent ocular conditions. Additional exclusion criteria encompassed a history of ocular or periocular malignancies, active or suspected infections, complicated cataract surgery, a positive pregnancy test, or participation in a clinical trial involving investigational drugs within 30 days (or 5 half-lives of the investigational drug).

All patients followed the same postoperative medication regimen. This included one drop of ofloxacin administered six times daily for one week and one drop of corticosteroid administered six times daily, tapered over the course of one month.

The study consisted of a preoperative evaluation (T0) conducted before cataract surgery, followed by postoperative follow-ups at week one (T1), one month (T2), and three months (T3). At each visit, patients underwent thorough clinical evaluations and completed symptom assessment questionnaires. These included the Standard Patient Evaluation of Eye Dryness (SANDE) score and the Ocular Surface Disease Index (OSDI), administered three days before surgery and again at one month and three months postoperatively.

Clinical assessments included: biomicroscopy of the anterior segment, corneal and conjunctival staining with fluorescein, graded using the National Eye Institute/Industry (NEI) grading scale, Tear Break-Up Time (TBUT), Schirmer test (type 1), and Ocular Surface Disease Index (OSDI) score. Data were analyzed using two-way ANOVA to compare mean values across the different time points. Statistical analyses were performed using GraphPad software version 10.4.1. A *p*-value < 0.05 was considered statistically significant.

## 3. Results

Twenty (20) consecutive patients (11 females and 9 males, mean age 71.58) underwent cataract surgery. Despite achieving excellent visual outcomes (BCVA 20/20), a significant number of the patients reported ocular discomfort during the follow-up, highlighting the phenomenon of “unhappy 20/20”. The analysis focused on the progression of symptoms and clinical signs related to ocular surface dysfunction, comparing values from the preoperative period (T0), one month postoperatively (T1), and three months postoperatively (T2).

**Figure 1:**
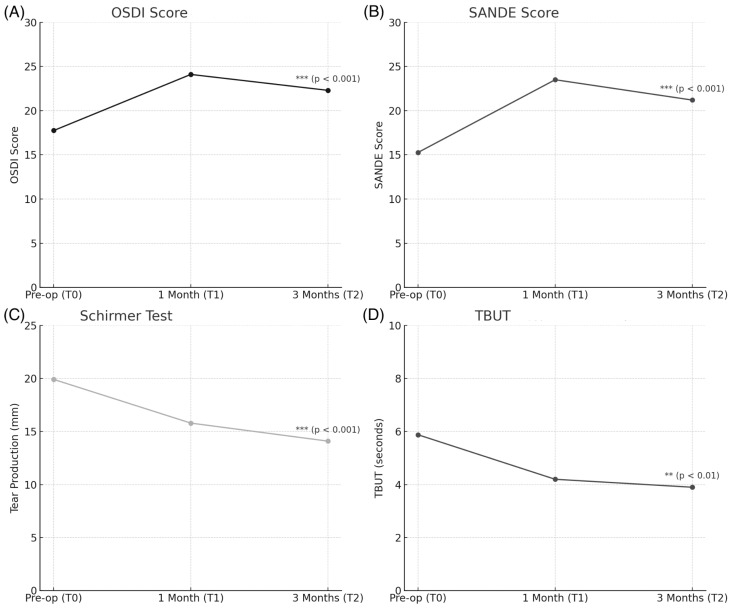
Time-related changes in ocular surface parameters preoperatively (T0), one month postoperatively (T1), and three months postoperatively (T2). (**A**) OSDI Scores: The mean OSDI score showed a significant increase postoperatively, indicating worsening symptoms of ocular surface disease. Statistical significance was confirmed with a *p*-value < 0.001 (***). (**B**) SANDE Scores: Postoperative SANDE scores similarly demonstrated a significant increase, highlighting worsening dry eye symptoms. The observed change was statistically significant with a *p*-value < 0.001 (***). (**C**) Schirmer Test Results: A decrease in tear production was noted over time, with statistical significance (*p*-value < 0.001, ***), indicating reduced tear production post surgery. (**D**) TBUT (Tear Break-Up Time): Tear film stability decreased postoperatively, with a statistically significant change (*p*-value < 0.01, **). Asterisks next to data points denote statistical significance: ** *p* < 0.01, *** *p* < 0.001.

**Figure 2:**
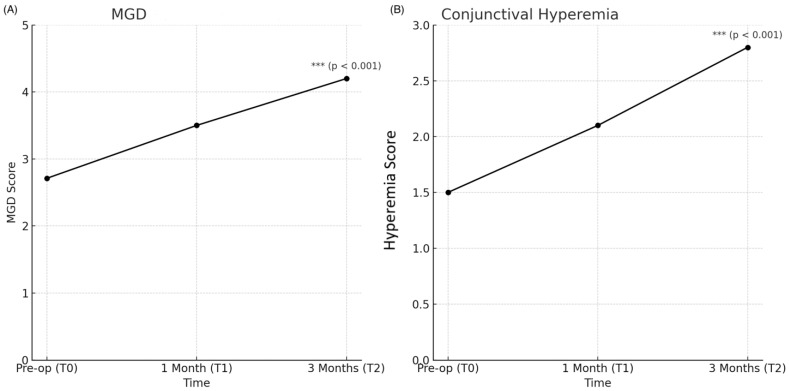
Time-related changes in ocular surface parameters preoperatively (T0), one month postoperatively (T1), and three months postoperatively (T2). (**A**) MGD: Meibomian gland dysfunction severity increased postoperatively. Statistical significance was confirmed with a *p*-value < 0.001 (***). (**B**) Conjunctival Hyperemia (redness of the conjunctiva): Postoperative conjunctival hyperemia demonstrated a significant increase. The observed change was statistically significant with a *p*-value < 0.001 (***). Asterisks next to data points denote statistical significance: *** *p* < 0.001.

The mean preoperative visual acuity was 0.22 logMar (95% CI: 0.10–0.34). For the OSDI (Ocular Surface Disease Index), the preoperative mean score was 17.75 ± 18.16 (mean ± SD), showing substantial variability across patients. Postoperatively, OSDI scores increased, reflecting worsening symptoms after surgery. ANOVA revealed a significant effect of time on OSDI scores (F(5, 138) = 17.64, *p* < 0.001), indicating that ocular surface symptoms significantly worsened postoperatively (Figure 1A). For SANDE (Symptom Assessment in Dry Eye), the preoperative mean score was 15.27 ± 12.41. ANOVA confirmed a significant interaction between time and SANDE scores (F(5, 138) = 12.00, *p* < 0.001) further demonstrating the worsening of dry eye symptoms post surgery (see Figure 1B). For the Schirmer test (Type I, measuring tear production), the preoperative mean score was 19.92 ± 10.06 (MEAN ± SD). ANOVA results confirmed a significant reduction in tear production between T0 and T2 (F(5, 138) = 17.64, *p* < 0.001), indicating decreased tear function postoperatively (Figure 1C). For the T-BUT (Tear Break-Up Time, measuring tear film stability), the preoperative mean was 5.88 ± 2.64 s (mean ± SD). Postoperative decline in tear film stability was confirmed by ANOVA, with a significant effect of time on T-BUT scores (F(5, 138) = 12.00, *p* < 0.001) (Figure 1C). For meibomian gland dysfunction (MGD), the preoperative mean score was 2.71 ± 0.69 (mean ± SD). ANOVA indicated a significant increase in MGD severity postoperatively (F(5, 138) = 17.64, *p* < 0.001), contributing to the worsening of ocular surface symptoms (Figure 2A). For conjunctival hyperemia (the redness of the conjunctiva), the preoperative mean was 1.50 ± 1.18 (mean ± SD). The increase in conjunctival hyperemia postoperatively was statistically significant, with ANOVA confirming the time effect (F(5, 138) = 12.00, *p* < 0.001) (Figure 2B).

**Figure 3:**
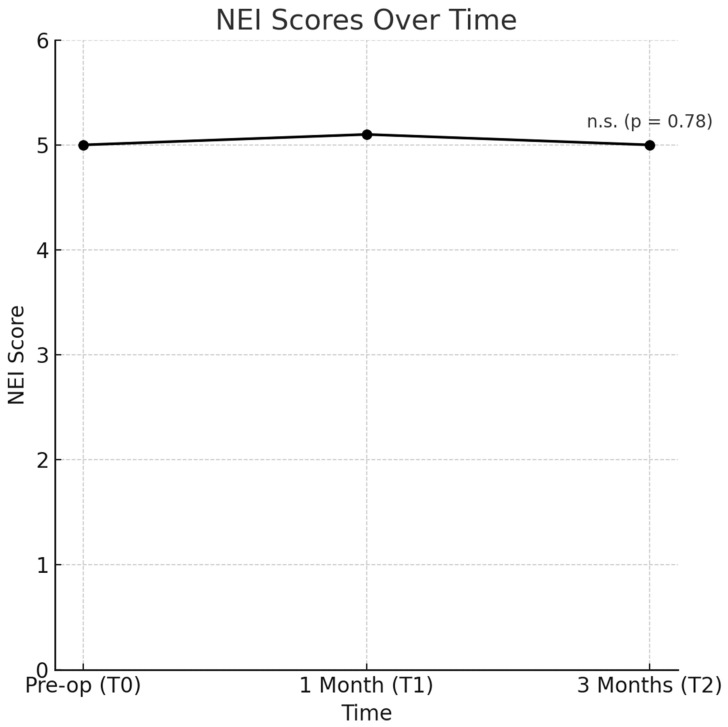
NEI score between the preoperative time point (T0), the 1-month follow-up (T1), and the 3-month follow-up (T2) did not reveal any statistically significant changes.

The analysis of the NEI score between the preoperative time point (T0), the 1-month follow-up (T1), and the 3-month follow-up (T2) did not reveal any statistically significant differences (F = 0.26, *p* = 0.78), indicating that overall ocular health, as measured by the NEI score, remained stable over time (Figure 3).

Despite achieving favorable visual outcomes, patients exhibited a significant increase in ocular surface dysfunction following cataract surgery, as evidenced by worsening scores in both symptom questionnaires and functional markers.

## 4. Discussion

Postsurgical OSSF affects 44% of cataract surgery patients. It appears that the local homeostatic mechanism is compromised due to neurogenic damage [5]. This neurogenic dysregulation and inflammation result in ocular surface protective system failure. Surgical or inflammatory trauma exacerbates dysregulation of para-inflammatory mechanisms, leading to chronic clinical inflammation [6]. Neuropeptides can generate neurogenic inflammation of the ocular surface, causing dry eye disease and OSSF. Our study showed that ocular surface markers—specifically, the NEI score, BUT, and Schirmer test—were already altered preoperatively in patients reporting ocular surface discomfort following surgery. Symptoms included conjunctival hyperemia, ocular surface irritation, itching, tearing, and foreign body sensation, which can persist for over six months post-cataract surgery, significantly impacting quality of life. This discomfort makes patients “unhappy 20/20” despite successful surgery. This is particularly common in patients with preoperative subclinical or clinical dry eye. Specifically, 80.9% of patients reported post-cataract ocular surface discomfort (OSD), with more than 60% of unmanaged cases suffering from OSD preoperatively, ultimately leaving 35% of patients dissatisfied [2]. Ishrat et al. reported clinical signs of ocular surface discomfort in 9% of patients 4 weeks after surgery [7], while Miyake and Yokoi documented such problems in 31% at the same time period [8]. In a study by Gupta et al., all 100 patients showed abnormalities in TBUT, Schirmer tests, and OSD symptoms at 12 weeks post surgery [9]. Another study by Choi et al. found persistent symptoms in 27% of patients at three months, as indicated by OSDI scores and reduced TBUT, increased corneal fluorescein staining, and meibomian gland dropout [10]. Ocular surface postsurgery failure (OSSF) is characterized by the deterioration of visual acuity several weeks after surgery that temporarily improves with the use of eye lubricants and it lasts almost for 6 months. The incidence of OSSF in cataract surgery candidates who are asymptomatic is higher than previously thought. In one study [2], upwards of 60% of routine cataract patients were asymptomatic, yet 50% had central corneal staining. In another study [7], the incidence of OSD in patients presenting for cataract surgery was over 80%, and in those who were asymptomatic, over 50% had an abnormal tear osmolarity or matrixmetalloproteinase-9 (MMP-9) level [11].

Aberrations induced by OSSF compromise visual quality through the more complex optics associated with multifocal technology [12]. Patient expectations from their cataract surgery have been high through the years and costs have been increased to new levels through the use of premium lens technology [13,14]. By routinely screening for OSD surgeons will be able to improve outcomes with premium IOLs. Not diagnosing and treating OSD prior to surgery lead to an increased risk of patient’s dissatisfaction [15]. Numerous studies highlight the importance of preoperative screening to identify preexisting ocular surface conditions, enabling targeted treatment to reduce the risk of intraoperative and postoperative complications. A systematic algorithm, such as the one proposed by Chiang et al. [16], could enhance these strategies by providing a tailored approach based on comprehensive preoperative assessment and risk stratification. These measures not only improve ocular surface management but also optimize postoperative visual outcomes. Intraoperatively, it is essential to adopt surgical techniques that minimize ocular trauma, such as small-incision cataract surgery and phacoemulsification, rather than extracapsular cataract extraction (ECCE) [17,18,19]. Surgeons should also consider reducing incision size, operative time, irrigation, and exposure to microscopic light. Additionally, awareness of potential risks associated with femtosecond laser techniques and aspirating speculums, particularly in patients predisposed to DED, is paramount. Postoperatively, early screening and continuous monitoring for dry-eye disease and ocular surface complications are crucial, especially in high-risk patients, such as those with GVHD or Stevens–Johnson syndrome, who are susceptible to severe complications. Close follow-up is necessary to monitor for recurrence of symptoms or the development of complications, ensuring a dynamic approach to patient care. In this context, implementing a comprehensive algorithm as described by Chiang et al. may provide a structured framework to improve decision-making in preoperative screening and surgical planning, ultimately leading to better patient outcomes [16]. Treatment plans should be tailored based on the type and severity of DED. The use of preserved eye drops free during and after surgery may affects the ocular surface with injury to corneal epithelial and conjunctival epithelial and goblet cells [4]. Li et al. investigated 37 patients before and up to 3 months after surgery and documented ocular surface discomfort after surgery in most, with the presence of conjunctival epithelial squamous metaplasia on impression cytology, especially in the region of the lower lid [20]. It is important to highlight that the use of preservatives can adversely affect the ocular surface. Prolonged use of low concentrations of preservatives can have side effects, while high concentrations of some preservatives can cause immediate damage and irritation to eye tissue [21].

The impact of OSD on topography, biometry, keratometry, and higher-order aberrations is one of the major causes of disappointing postoperative outcomes [11]. As prevention of OSD a preoperative intervention can be made, preservative free lipophilic artificial tears remain the initial therapy for all forms, but eventually, they may not be sufficient [22]. In case of MGD, eyelid hygiene is fundamental. A prospective case series by Han et al. showed that following cataract surgery there are increased lid margin abnormalities at 3 months [23]. Pre-operative management of MGD is important before cataract surgery. Treatment regimens for MGD include the regular use of warm compressors, lid hygiene, treatment of demodex, and the administration of systemic tetracycline antibiotics and topical azithromycin [24,25]. In cases where inflammation is not sufficiently reduced by the usual regimen, a short-term regimen of non-preserved low-level steroids, have consistently produced encouraging outcomes, and may be proposed as a adjunctive therapy for highly moderate to severe cases [26]. Patients should be instructed to postoperatively continue the preoperative regimen. As part of the overall surgical plan, the postoperative eyedrop regimen should be carefully considered in patients with OSD. Postoperative drops, in particular, those with preservatives, might lead to toxicity and exacerbations of OSD [22]. When possible, the most epithelio-toxic antibiotics should be avoided, or used with caution and/or for a short duration. More frequent follow-up is usually required to ensure ocular surface compatibility and to determine if a change in treatment is necessary [27]. If ocular surface inflammation is severely worsened, some patients may require more aggressive treatment, including short-term topical (unpreserved) steroid therapy, or even topical immunomodulatory drugs [11]. Our analysis underscores the importance of considering the type of surgical intervention as a potential factor influencing postoperative outcomes. Although the present study focused on a homogeneous cohort undergoing standard phacoemulsification, future investigations should explore the differential effects of various surgical techniques, such as femtosecond laser-assisted cataract surgery (FLACS) or micro-incision cataract surgery (MICS), on ocular surface parameters. This stratified approach could provide deeper insights into how surgical variations impact the development and progression of ocular surface dysfunction. Such findings would inform tailored surgical planning and perioperative management strategies, ultimately improving both objective outcomes and patient satisfaction. In addition to the factors assessed in this study, other potential risk factors for postoperative dry eye warrant consideration. Smoking, diabetes mellitus, high scores on the Hospital Anxiety and Depression Scale, and longer surgical incision length have all been identified as contributors to postoperative ocular surface dysfunction. Future studies should incorporate these variables to provide a more comprehensive understanding of their impact on postoperative dry eye and patient satisfaction. To address the dissatisfaction experienced by ‘unhappy 20/20’ patients, a multimodal approach is needed. This could include comprehensive preoperative evaluations to identify risk factors for poor visual quality, tailored patient counseling to set realistic expectations, and targeted postoperative interventions, such as enhanced ocular surface management and treatment of subtle optical aberrations. These strategies may help optimize outcomes and improve patient satisfaction. In conclusion, the establishment of appropriate preoperative and postoperative care protocols is paramount to minimizing the risk of ocular surface dysfunction and achieving excellent visual outcomes while ensuring patient satisfaction. Effective management of postoperative inflammation is equally critical, including the use of long-term, preservative-free, lipophilic lubricants and the avoidance of epitheliotoxic drugs. Implementing these strategies can help preserve or restore ocular surface homeostasis, thereby reducing the likelihood of ocular discomfort even after a well-executed surgical procedure. By prioritizing these measures, we can optimize both clinical outcomes and the overall patient experience.

## Data Availability

The data supporting the findings of this study are not publicly available due to patient privacy and confidentiality regulations.
